# Classification of pediatric dental diseases from panoramic radiographs using natural language transformer and deep learning models

**DOI:** 10.3389/frai.2026.1754498

**Published:** 2026-03-03

**Authors:** Tuan D. Pham, Seba Al-Hebshi

**Affiliations:** Barts and The London School of Medicine and Dentistry, Queen Mary University of London, London, United Kingdom

**Keywords:** artificial intelligence, children, deep learning, dental diseases, natural language processing, panoramic radiographs

## Abstract

**Introduction:**

Accurate classification of pediatric dental diseases from panoramic radiographs is essential for early diagnosis and effective treatment planning. While deep learning models traditionally operate directly on image data, text-based representations generated from radiographs may provide an alternative strategy for disease classification.

**Methods:**

This study proposed a text-driven framework in which a natural language transformer was used to generate structured textual descriptions from panoramic radiographs. These descriptions were subsequently classified for binary disease detection using three deep learning architectures: a one-dimensional convolutional neural network (1D-CNN), a long short-term memory (LSTM) network, and a pretrained Bidirectional Encoder Representations from Transformer (BERT) model. Model performance was evaluated and compared against three pretrained convolutional neural networks trained directly on radiographic images.

**Results:**

The 1D-CNN achieved the highest performance with 84% accuracy, demonstrating balanced classification across disease categories. The BERT model reached 77% accuracy, showing strong performance in detecting periapical infections but comparatively lower sensitivity for caries identification. The LSTM model performed substantially worse, achieving 57% accuracy. Both the 1D-CNN and BERT text-based approaches outperformed the three image-based pretrained CNN models.

**Discussion:**

These findings suggest that text-based classification of panoramic radiographs is a potential alternative to conventional image-based deep learning methods. Language-driven models show promise for radiographic interpretation; however, challenges remain in achieving consistent generalizability across disease types. Future research should focus on improving radiograph-to-text generation quality, developing hybrid architectures that integrate textual and visual features, and validating performance on larger and more diverse datasets to strengthen clinical applicability.

## 1 Introduction

Accurate and early diagnosis of pediatric dental diseases is essential for effective treatment planning and the prevention of long-term complications (Muntean et al., 2015; Zou et al., 2018; Oral Health in America: Advances and Challenges, 2021; Featherstone et al., 2021; Abdelaziz, 2023). Panoramic radiographs are widely used in dentistry to assess conditions such as caries and periapical infections. Traditionally, diagnosis relies on manual interpretation by dental professionals, a process that can be time-consuming and subject to interobserver variability. To enhance diagnostic efficiency and accuracy, artificial intelligence (AI) has emerged as a promising tool in dental imaging, particularly through deep learning models trained for automated disease classification (Ossowska et al., 2022; Ding et al., 2023; Dhingra, 2023; Ghaffari et al., 2024).

Recent advances in deep learning have enabled AI models to assist in detecting and diagnosing a range of pediatric dental conditions, an area that has historically received less attention than adult dental diagnostics. Kaya et al. (2022) introduced a deep learning model specifically designed to detect permanent tooth germs in pediatric panoramic radiographs, addressing a gap in prior research, which has primarily focused on adult radiographs. Similarly, Ong et al. (2024) developed a fully automated deep learning framework for assessing dental development stages in pediatric radiographs, further demonstrating AI's potential to improve diagnostic accuracy and clinical decision-making.

The systematic review by Khanagar et al. (2022) highlights the transformative impact of AI in pediatric dentistry, particularly in early disease detection, which is critical for timely intervention and treatment. Their review examined various AI models applied in diagnosing conditions such as dental caries and periodontal diseases, reinforcing the potential of machine learning in pediatric oral healthcare. Beyond disease detection, AI also plays a role in treatment planning and patient management. La Rosa et al. (2024) explored how AI algorithms can identify early signs of dental pathologies and improve orthodontic diagnoses through automated image analysis. This aligns with Mahajan et al. (2023), who conducted a systematic review on the effectiveness of AI in pediatric dentistry, concluding that AI-based diagnostic tools have demonstrated promising results in clinical applications.

Despite these advancements, the implementation of AI in pediatric dentistry faces several challenges, particularly concerning data quality, dataset size, and model generalizability. AI models require large and diverse datasets to achieve robust performance, yet pediatric dental datasets are often limited, leading to potential biases and decreased model reliability. Hsieh (2024) emphasized that while AI holds great promise for comprehensive dental disease classification, challenges such as class imbalance and the rarity of certain conditions can hinder model training and diagnostic accuracy. These limitations underscore the need for ongoing research to refine AI methodologies, improve data preprocessing techniques, and develop strategies for overcoming data scarcity.

The integration of AI into pediatric dental disease classification from panoramic radiographs is a rapidly evolving field with significant potential to enhance diagnostic accuracy, reduce human error, and improve patient outcomes. However, to fully realize its benefits, further research is needed to address challenges related to data availability, annotation consistency, and model validation across diverse populations. While AI-driven image-based classification using convolutional neural networks (CNNs) has demonstrated success, such approaches often require extensive preprocessing as well as large datasets to achieve high performance. Hybrid models that integrate text descriptions from images and deep learning for classification could provide a promising direction for improving accuracy, interpretability, and scalability in pediatric dental diagnostics.

Large language models (LLMs), particularly ChatGPT, have emerged as transformative tools in medical and dental applications, leveraging advanced natural language processing (NLP) capabilities to enhance various aspects of healthcare delivery. These models are designed to understand and generate human-like text, making them valuable for a range of tasks, including patient communication, clinical decision support, and medical education.

In addition to these applications, LLMs enable the transformation of visual medical information into structured textual representations. To the best of the authors' knowledge, text-based classification of dental images has not been previously reported, and there is currently no established literature directly addressing this paradigm. Consequently, a broader discussion of LLMs in dentistry and healthcare is provided to establish the necessary background and to contextualize the proposed approach within the emerging landscape of language-driven medical AI applications.

One of the primary applications of ChatGPT in healthcare is its ability to assist in clinical decision-making. A study by Giannakopoulos et al. (2023) evaluated the performance of ChatGPT and other LLMs in supporting evidence-based dentistry, demonstrating that these models can effectively provide relevant information and aid practitioners in making informed decisions, ultimately improving patient care. Similarly, Huang et al. (2023) highlighted ChatGPT's potential in dental education, suggesting that it could serve as a valuable resource for students by offering instant access to information and facilitating interactive learning experiences.

In patient communication, LLMs like ChatGPT have shown promise in addressing inquiries and enhancing patient satisfaction. A survey conducted by Yong et al. (2024) found that LLMs were effective in resolving patient complaints, emphasizing their ability to generate thoughtful and contextually appropriate responses. This capability is particularly valuable in fostering better patient-provider relationships and improving overall healthcare experiences.

The integration of LLMs in medical education has also been a focal point of recent research. Abd-Alrazaq et al. (2023) explored how these models enhance medical students' learning experiences by providing comprehensive explanations of complex medical concepts. Similarly, Wu et al. (2024) evaluated ChatGPT's performance on nursing licensure examinations, indicating that LLMs can serve as effective study aids, potentially improving educational outcomes.

Despite these advantages, the deployment of LLMs in healthcare is not without challenges. Lin et al. (2024) noted that while ChatGPT offers valuable support in critical care medicine, it also has limitations, such as the potential to generate inaccurate or misleading information. This concern is echoed by Tiwari et al. (2024), who conducted a systematic review on the implications of ChatGPT in public health dentistry, highlighting the need for rigorous assessment of risks, including biases and misinformation, before widespread adoption.

The motivation for this study stems from the need to enhance the automated classification of pediatric dental diseases from panoramic radiographs by leveraging both NLP and deep learning. Traditional image-based classification methods often require extensive preprocessing, high-resolution datasets, and expertise to interpret complex radiographic features. To overcome these challenges, this study explores an alternative approach by using ChatGPT to generate textual descriptions of the conditions depicted in panoramic radiographs. These textual representations serve as input for deep learning models, enabling disease classification through language-based analysis rather than direct image interpretation. This approach aims to improve diagnostic accuracy, enhance model interpretability, and reduce reliance on large-scale labeled radiographic datasets. By integrating AI-driven text generation with deep learning classification, this study seeks to demonstrate the feasibility of a text-based framework for dental disease diagnosis, offering a scalable and interpretable alternative to traditional radiographic analysis.

## 2 Methods

### 2.1 Panoramic radiographs

The children's dental panoramic radiographs dataset (Zhang et al., 2023), hosted on Figshare (Zhang et al., 2023), is utilized in this study. The dataset, titled *Child Dental Disease Detection Dataset*, was originally designed with a structured division into “Train” and “Test” subsets, as described in Zhang et al. (2023). Each subset contains both the original radiographic images and corresponding expert annotations. The dataset consists of 100 images categorized into five dental diseases: caries (class 1), periapical infections (class 2), pulpitis (class 3), deep sulcus (class 4), and dental developmental abnormalities (class 5).

The annotation process involved six dental experts to ensure accuracy and reliability. Four experts independently annotated 25 randomly assigned anonymous images in the first round. Two additional experts then reviewed the labeled images to assess annotation accuracy. In cases of ambiguity, all six experts engaged in a consensus discussion, and the final labels were determined based on their unified decision. This multi-step validation process aimed to enhance the consistency and diagnostic precision of the dataset annotations. For this study, only class 1 (caries) and class 2 (periapical infections) were utilized to maintain a balanced dataset for machine learning classification. Thus, the final data subset used in this study consisted of 29 panoramic radiographs for dental caries and 29 for periapical infections, resulting in a total of 58 images. This selection was made to ensure class balance and to support a more reliable evaluation of the machine learning models.

### 2.2 LLM: ChatGPT

ChatGPT (OpenAI, 2023) is an advanced LLM based on OpenAI's GPT architecture, designed to process and generate human-like text by leveraging deep learning techniques. Trained on vast datasets, including general knowledge, medical literature, and structured language patterns, ChatGPT can understand, summarize, and generate contextual responses across diverse domains. Its transformer-based architecture allows it to analyze complex relationships between words, enabling it to generate coherent and contextually relevant descriptions. This makes ChatGPT a valuable tool in medical AI applications, particularly for interpreting and summarizing clinical information.

Although ChatGPT does not directly analyze images, it can process structured input derived from automated or human-interpreted imaging analyses. By leveraging pre-existing medical knowledge, it can describe abnormalities such as bone loss, periapical radiolucencies, or carious lesions based on textual cues extracted from images. High-resolution panoramic radiographs contain detailed anatomical structures, but reduced-resolution images may lose some fine details. ChatGPT can compensate for this by generating structured descriptions that highlight key diagnostic features, ensuring that relevant clinical insights are preserved for downstream classification tasks.

A text-based approach provides an interpretable summary of radiographic findings, making AI-driven diagnostic models more accessible to clinicians. ChatGPT-generated descriptions allow for a structured, standardized representation of dental disease characteristics, improving transparency in AI-assisted diagnosis. Additionally, processing full-resolution radiographs with deep learning models requires substantial computational resources. By reducing image resolution and transforming key features into text, ChatGPT enables a more computationally efficient pipeline while retaining essential diagnostic information. By converting panoramic radiograph features into text, ChatGPT serves as a bridge between image interpretation and deep learning classification, offering an efficient, interpretable, and scalable approach to dental disease diagnosis.

To ensure consistency and reproducibility in the generation of textual descriptions, the same prompt structure was applied to all panoramic radiographs. Each image was processed independently in a single-turn interaction, without iterative refinement, conversational feedback, or manual post-editing of the generated output. The objective was to obtain structured, standardized, and non-interpretative textual representations of observable visual content rather than clinically validated diagnoses.

The full prompt template used for all images was as follows:

*You are given a dental panoramic radiograph. Describe only observable anatomical and radiographic features. Do not provide diagnosis, severity, or clinical interpretation. Do not speculate or infer disease. Use neutral medical language. Write exactly 12 words. Return only the sentence, with no extra text*.

To minimize subjectivity and hallucination, the prompt explicitly prohibited diagnostic inference, severity assessment, and speculative language. The generated textual descriptions were subsequently reviewed by a qualified dentist to assess their anatomical plausibility, consistency with the source images, and adherence to the non-interpretative prompt constraints. This assessment was qualitative in nature and aimed to identify gross inconsistencies or clinically implausible descriptions rather than to provide formal diagnostic validation.

### 2.3 LSTM

LSTM networks (Hochreiter and Schmidhuber, 1997) are a type of recurrent neural network (RNN) designed to handle sequential data by addressing the issue of vanishing gradients, which commonly affects traditional RNNs. LSTMs are particularly effective in learning long-range dependencies, making them well-suited for tasks involving time series, NLP, and speech recognition.

Unlike standard RNNs, which struggle to retain information over long sequences, LSTMs use a specialized gating mechanism to regulate the flow of information. The network consists of three key gates: the forget gate, which decides what information to discard from the previous state; the input gate, which determines what new information to store; and the output gate, which controls what information is passed to the next time step. These gates allow LSTMs to selectively remember or forget information, enabling them to capture dependencies across long sequences while mitigating issues related to gradient decay.

**Figure 1:**
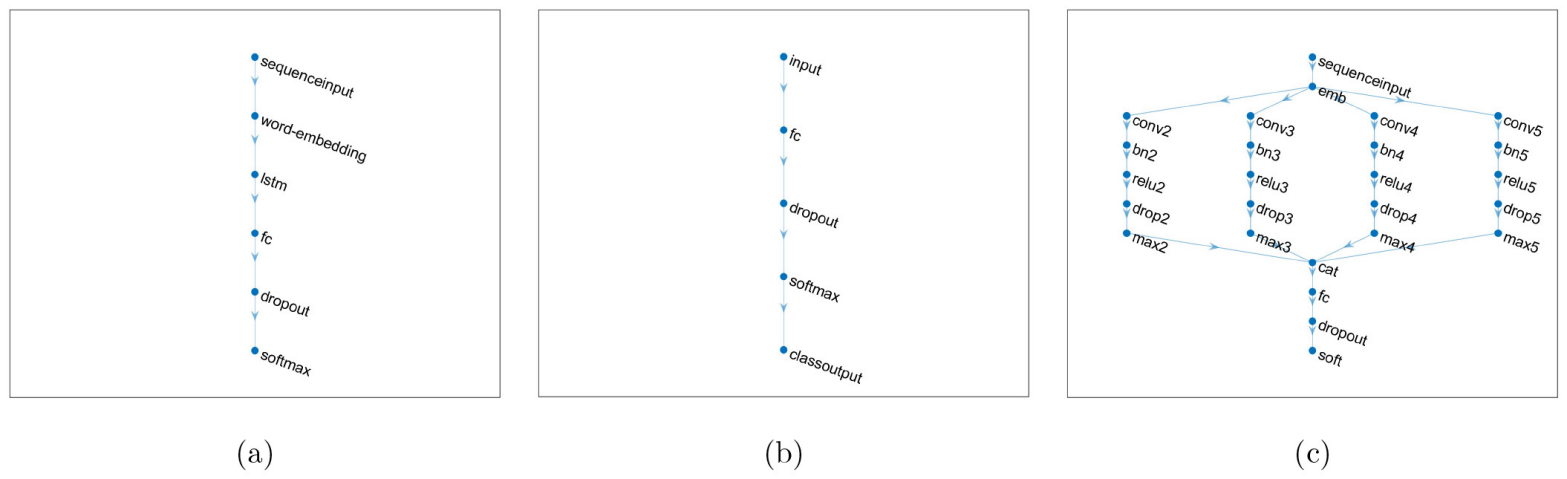
Network layers: LSTM **(a)**, model trained with features extracted from BERT **(b)**, and 1D-CNN **(c)**.

The architecture of the LSTM model used in this study consists of a sequence input layer with an input size of 1, followed by a word embedding layer with an embedding dimension of 50. An LSTM layer with 100 hidden units was included, with the output mode set to “last” to process the entire sequence. A fully connected layer was added, with the number of units matching the number of classes in the data. A dropout layer with a rate of 0.2 was applied to prevent overfitting, followed by a softmax layer to classify the input into one of the two classes. Figure 1a shows the architecture of the LSTM.

For training, the Adam optimizer and cross-entropy loss were chosen, and the maximum number of epochs was set to 600. The batch size was specified as 128, and the model was evaluated using accuracy. Validation data was provided, and the best network based on validation performance was saved.

### 2.4 BERT

BERT (Devlin et al., 2019) is a deep learning model, designed to process and understand natural language with high contextual awareness. Unlike traditional word embedding techniques that represent words independently, BERT captures deep bidirectional contextual relationships by considering both preceding and following words in a sentence. This ability makes it particularly powerful for tasks such as text classification, question answering, and language inference.

BERT is based on the transformer architecture, which relies on self-attention mechanisms to process entire sequences in parallel rather than sequentially, as in RNNs and LSTM networks. The model is pretrained on large text corpora using two key objectives: masked language modeling, where random words are masked, and the model learns to predict them, and next sentence prediction, where the model learns relationships between sentence pairs. This pretraining enables BERT to generalize well to various NLP tasks after fine-tuning on domain-specific datasets.

In this study, the BERT-Base model, consisting of 108.8 million learnable parameters, was used for classification. A tokenizer was employed to encode the text into sequences of integers, enabling the model to process the input effectively. Following tokenization, the data was partitioned into training and validation sets as previously described. To prepare the data for training, the BERT tokens were organized into mini-batches. This step ensures that the data is processed in manageable chunks, which is crucial for training large models like BERT. The BERT model was then used to transform the tokenized data into feature vectors by extracting embeddings, which served as input features for both the training and validation datasets.

Subsequently, a deep learning network was constructed for classification. The network architecture included a feature input layer, a fully connected layer to map the feature vectors to class labels, a dropout layer to reduce overfitting through regularization, and a softmax layer to output the class probabilities. This network was specifically designed to classify the BERT-derived feature vectors. Figure 1b shows the architecture of the network designed for classifying BERT-derived feature vectors, referred to as BERT for brevity.

The training process was configured with the following options: a mini-batch size of 128, the Adam optimizer, a maximum of 600 epochs, an initial learning rate of 0.0001, and without data shuffling to ensure consistent data order during training.

### 2.5 1D-CNN

For classification using a 1D-CNN (LeCun et al., 1998), the network architecture was designed for text classification and begins with specifying the input size as 1, which corresponds to the channel dimension of the input integer sequence. The input data were then embedded using a word embedding layer with a dimension of 50.

The next step involved creating blocks of layers for different *n*-gram lengths, specifically 2, 3, 4, and 5. Each block consists of a 1D convolutional layer, batch normalization, a ReLU activation layer, a dropout layer with a rate of 0.2, and a global max pooling layer. For each block, 100 convolutional filters were used, and the size of the convolutional filter corresponds to the *n*-gram length. These blocks were connected to the word embedding layer, and their outputs were concatenated using a concatenation layer.

The final part of the architecture includes a fully connected layer, which outputs the class predictions, followed by a softmax layer for classification. The network was structured to handle various *n*-gram lengths by connecting each block to the word embedding layer and finally linking the pooling layers to the concatenation layer. Figure 1c shows the architecture of the 1D-CNN.

The network was trained using the Adam optimizer with a mini-batch size of 128. The model was optimized using cross-entropy loss, The training included validation using a separate validation dataset. The network with the lowest validation loss was saved.

### 2.6 Binary classification performance metrics

In this binary classification task, the objective is to distinguish between caries and periapical infections. The classification parameters are defined as follows: true positive (TP) refers to periapical infections correctly identified as periapical infections, while false positive (FP) denotes caries incorrectly classified as periapical infections. True negative (TN) represents caries accurately identified as caries, and false negative (FN) refers to periapical infections incorrectly classified as caries.

The performance of the classification model is evaluated using several key metrics. Accuracy (ACC) measures the proportion of correct predictions, including both true positives and true negatives, relative to the total number of predictions for the two classes. Sensitivity (SEN) reflects the model's ability to correctly identify periapical infections, while specificity (SPE) assesses its ability to accurately classify caries. Precision (PRE) quantifies the proportion of cases predicted as periapical infections that actually belong to the class of periapical infection.

The F1 score is a metric that provides the harmonic mean of precision and sensitivity, balancing the trade-off between the two. It emphasizes the correct identification of periapical infections (true positives) while accounting for the impact of false positives and false negatives.

The area under the receiver operating characteristic curve (AUC) offers a comprehensive measure of the model's ability to separate the two classes. It represents the relationship between sensitivity and specificity, with a higher AUC indicating better performance and greater robustness in distinguishing between caries and periapical infections.

Mathematical expressions for these performance metrics are provided in Table 1.

**Table 1:** Mathematical expressions for metrics evaluating the binary classification model.

**Metric**	**Expression**
Accuracy (ACC)	$\frac{\text{TP}+\text{TN}}{\text{TP}+\text{TN}+\text{FP}+\text{FN}}$
Sensitivity (SEN)	$\frac{\text{TP}}{\text{TP}+\text{FN}}$
Specificity (SPE)	$\frac{\text{TN}}{\text{TN}+\text{FP}}$
Precision (PRE)	$\frac{\text{TP}}{\text{TP}+\text{FP}}$
F1 score	$\frac{2\text{TP}}{2\text{TP}+\text{FP}+\text{FN}}$

### 2.7 Hyperparameter selection and training configuration

Hyperparameters were selected using a fixed and reproducible configuration, consistent with the exploratory nature of this study and the limited dataset size. All neural network models were trained using the Adam optimiser with an initial learning rate of 0.0001 and a mini-batch size of 128, providing stable convergence across experiments. Models were trained for 100 epochs, with data shuffling applied at each epoch to minimize ordering effects. Validation accuracy was evaluated once per epoch to monitor training behavior.

The same training configuration was applied across all models to ensure methodological consistency and fair comparison. No adaptive or fold-specific hyperparameter optimisation was performed, as the emphasis of this work is on feasibility assessment and relative model behavior rather than performance maximization.

## 3 Results

**Figure 2:**
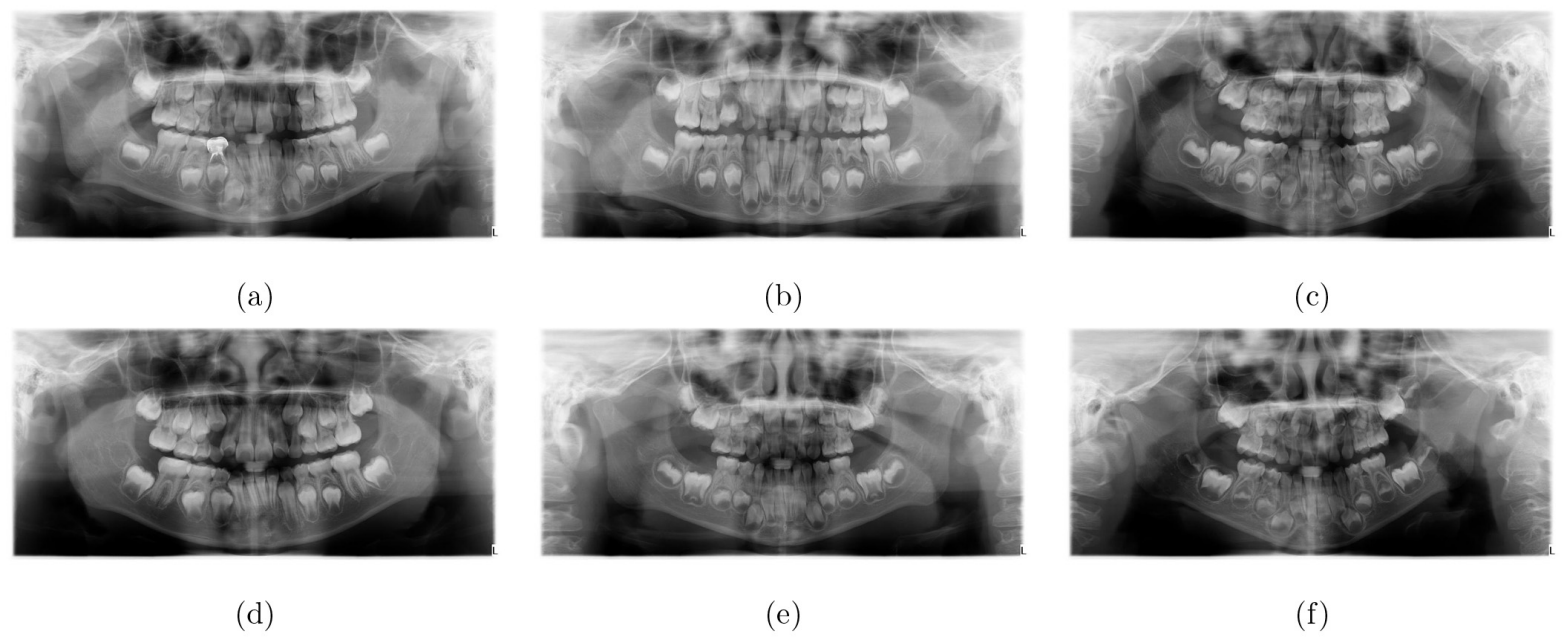
Reduced-resolution panoramic radiographs of pediatric dental diseases: caries or class 1 **(a–c)**, and periapical infections or class 2 **(d–f)**.

The resolution of the original panoramic radiographs was reduced 25 times. ChatGPT based on OpenAI's GPT-4 architecture was then used to generate concise, approximately 12-word descriptions of potential bone abnormalities depicted in the reduced-resolution images. Figure 2 displays reduced-resolution panoramic radiographs showing caries and periapical infections in children, while Table 2 presents the ChatGPT-generated descriptions of the bone abnormalities corresponding to these radiographs.

**Table 2:** ChatGPT-based descriptions of pediatric dental diseases on panoramic radiographs as shown in Figure 2.

**Figure 2**	**Class**	**Descriptions**
(a)	1	Possible bone loss, irregular jaw contours, fractures, cysts, or impacted teeth visible
(b)	1	Possible jaw asymmetry, unerupted teeth, bone resorption, cysts, fractures, or density irregularities
(c)	1	Impacted teeth with shortened roots, uneven cortical plates, and possible bone resorption
(d)	2	Impacted teeth, malocclusion, bone loss, cysts, fractures, asymmetry, infection, tumors, osteosclerosis, erosion, ankylosis, osteolysis
(e)	2	Dental crowding, impacted teeth, mandibular asymmetry, unerupted molars, root resorption, and hypercementosis
(f)	2	Impacted third molars, dental crowding, mandibular asymmetry, shortened roots, cystic changes, hypercementosis

For the processed text data, each document is converted into a sequence of numeric indices. To achieve this, a word encoding function was used to create a word encoding, which maps words to numeric indices. The documents were then converted into sequences, ensuring that all sequences are of equal length. The sequences were padded and truncated to a target length, and the longest sequence length was selected for uniformity.

**Figure 3:**
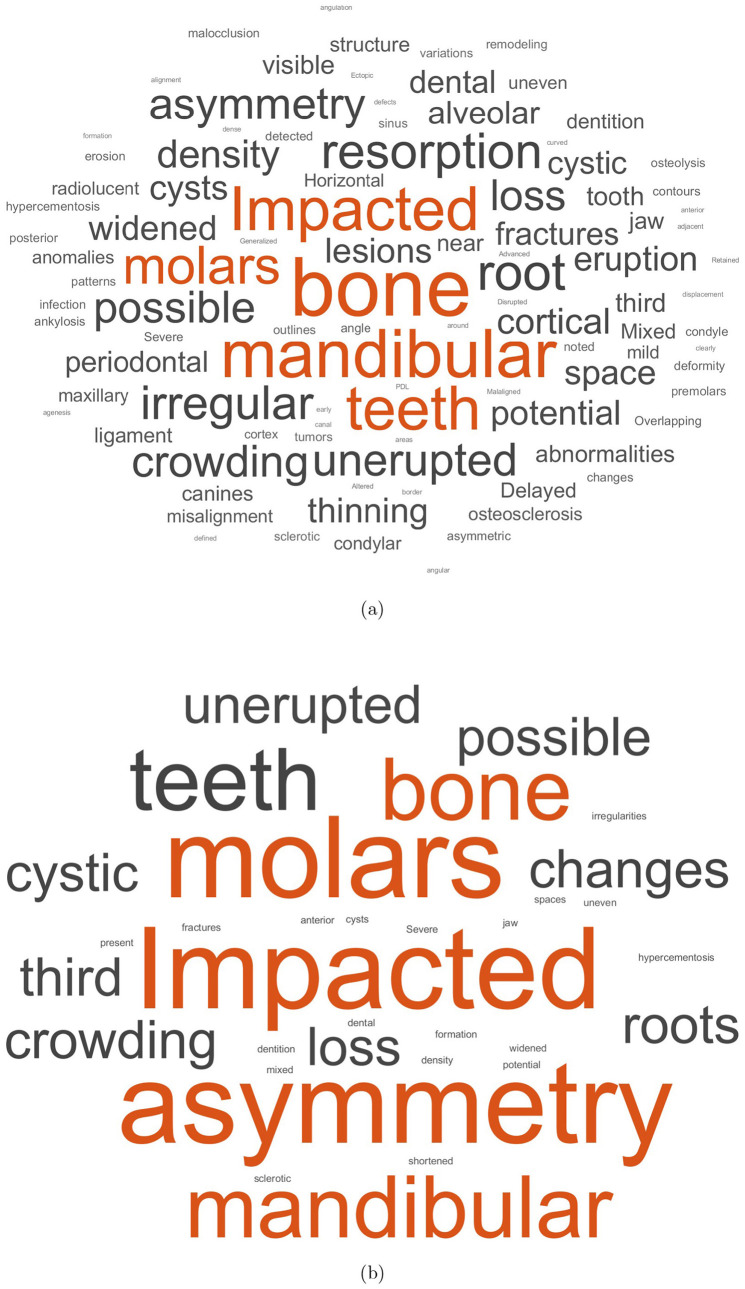
Word clouds of training **(a)** and test **(b)** data.

The text data were split into training and validation sets using a non-stratified holdout partition, where 90% of the data was allocated for training and the remaining 10% for validation. This partitioning process was repeated ten times, and the average classification results were recorded. Figure 3 shows the word clouds of training and test data.

A preprocessing function is defined to prepare the text data for model input. This function performs several tasks: it tokenizes the text, converts it to lowercase, and removes punctuation. The training and validation text data are then processed using this function.

To compare the text-based with image-based classifiers, three pretrained CNNs that are SqueezeNet (Iandola et al., 2016), GoogLeNet (Szegedy et al., 2015), and AlexNet (Krizhevsky et al., 2017) were also applied for the classification. The pretrained CNN models were trained and validated with reduced-resolution panoramic radiographs. Training options for the pretrained CNNs were specified as follows. The training process included data augmentation to enhance model generalization and prevent overfitting. Training images underwent random vertical flipping, translation of up to 30 pixels, and scaling variations between 90% and 110% to introduce variability and ensure the model learned good features rather than memorizing specific details. Validation images were resized without augmentation to maintain consistency during evaluation.

**Figure 4:**
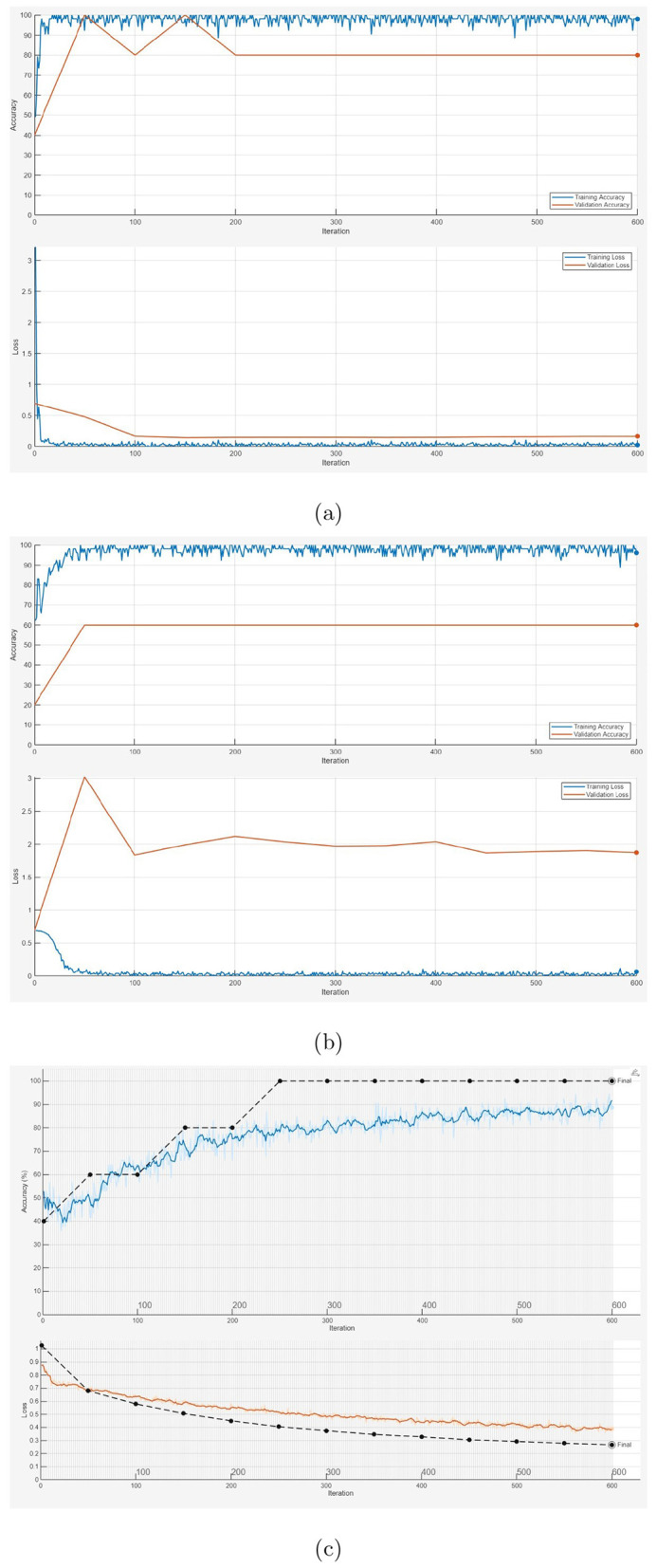
Training and validation processes: 1D-CNN **(a)**, LSTM **(b)**, and BERT feature based net, where solid and dotted lines indicate training and validation, respectively **(c)**.

**Figure 5:**
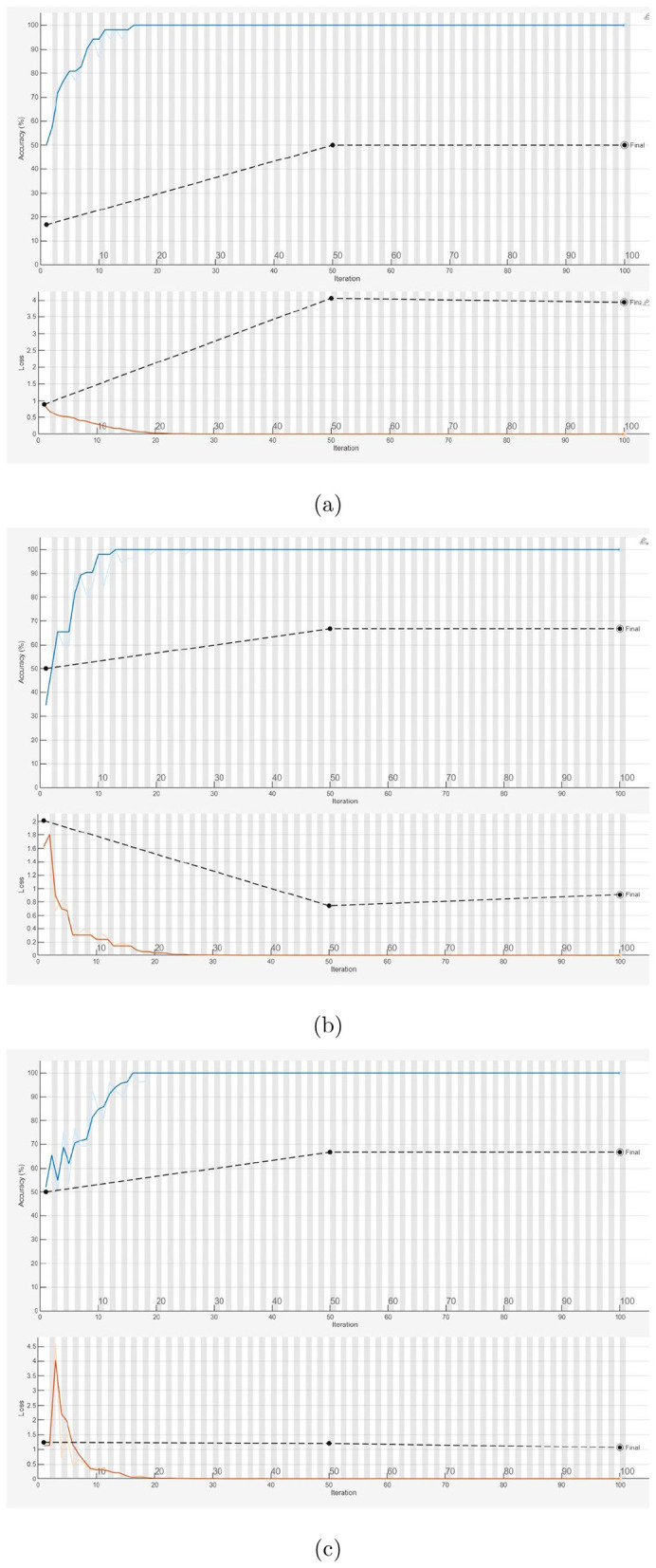
Training and validation processes of pretrained CNN models: SqueezeNet **(a)**, GoogLeNet **(b)**, and AlexNet **(c)**, where solid and dotted lines indicate training and validation, respectively.

Figure 4 illustrates the training and validation processes for the 1D-CNN, LSTM, and BERT models on a held-out partition. Figure 5 shows the training and validation processes for the three pretrained CNN models (SqueezeNet, GoogLeNet, and AlexNet) on a held-out partition. Table 3 presents the performance metrics obtained from the 1D-CNN, LSTM, BERT, and the three pretrained CNN models. To further assess the performance of the various AI models using images only (pretrained CNNs) and text only (1D-CNN, LSTM, and BERT), 5-fold cross-validation was performed. Tables 4, 5 report the performance metrics and the corresponding 95% confidence intervals estimated from the distribution of cross-validation scores, respectively.

**Table 3:** Performance measures of AI models evaluated using a 90% training and 10% testing split on reduced-resolution images and their corresponding derived textual representations.

**Model**	**ACC (%)**	**SEN (%)**	**SPE (%)**	**PRE (%)**	**F1**	**AUC**
**Input: images only**						
SqueezeNet	50.00	46.67	53.33	54.17	0.49	0.62
GoogLeNet	56.67	66.67	46.67	58.67	0.61	0.62
AlexNet	66.67	73.33	60.00	63.33	0.67	0.73
**Input: text only**						
LSTM	56.67	75.00	41.67	55.56	0.62	0.72
BERT	76.67	83.33	66.67	83.33	0.82	0.73
1D-CNN	84.00	86.67	86.67	86.67	0.84	0.93

**Table 4:** Cross-validated (5-fold) performance measures of AI models on reduced-resolution images and their corresponding derived textual representations.

**Model**	**ACC (%)**	**SEN (%)**	**SPE (%)**	**PRE (%)**	**F1**	**AUC**
**Input: images only**						
SqueezeNet	53.33 ± 4.56	46.67 ± 21.73	60.00 ± 19.00	53.43 ± 4.80	0.48 ± 0.15	0.54 ± 0.12
GoogLeNet	63.33 ± 7.45	63.33 ± 21.73	63.33 ± 21.73	68.33 ± 18.77	0.62 ± 0.11	0.71 ± 0.07
AlexNet	58.33 ± 5.89	76.67 ± 14.91	40.00 ± 19.00	56.78 ± 6.13	0.65 ± 0.05	0.63 ± 0.13
**Input: text only**						
LSTM	54.55 ± 8.11	62.00 ± 18.00	46.00 ± 13.00	53.97 ± 7.97	0.57 ± 0.12	0.62 ± 0.05
BERT	65.91 ± 1.52	77.50 ± 17.92	54.17 ± 15.96	62.29 ± 3.15	0.68 ± 0.06	0.74 ± 0.09
1D-CNN	75.61 ± 6.44	83.33 ± 11.79	66.67 ± 8.17	73.57 ± 6.21	0.78 ± 0.07	0.81 ± 0.12

**Table 5:** 95% confidence intervals for cross-validated (5-fold) performance measures of AI models using reduced-resolution images and their corresponding derived textual representations.

**Model**	**ACC (%)**	**SEN (%)**	**SPE (%)**	**PRE (%)**	**F1**	**AUC**
**Input: images only**						
SqueezeNet	[47.67, 59.00]	[19.68, 73.65]	[36.41, 83.60]	[47.47, 59.39]	[0.29, 0.66]	[0.39, 0.69]
GoogLeNet	[54.08, 72.59]	[36.35, 90.32]	[36.35, 90.32]	[45.02, 91.64]	[0.48, 0.76]	[0.62, 0.79]
AlexNet	[51.02, 65.65]	[58.16, 95.18]	[16.40, 63.60]	[49.17, 64.39]	[0.58, 0.71]	[0.47, 0.79]
**Input: text only**						
LSTM	[44.48, 64.61]	[39.41, 84.59]	[29.86, 62.14]	[44.07, 63.87]	[0.42, 0.72]	[0.56, 0.68]
BERT	[63.50, 68.32]	[48.98, 100]	[28.78, 79.56]	[57.29, 67.30]	[0.58, 0.79]	[0.60, 0.89]
1D-CNN	[67.61, 83.60]	[68.70, 97.97]	[56.53, 76.80]	[65.86, 81.28]	[0.69, 0.87]	[0.67, 0.96]

Given the exploratory nature of this study and the limited dataset size, all reported performance measures should be interpreted as preliminary. Rather than claiming definitive diagnostic performance, the results emphasizes relative model behavior, comparative trends, and feasibility across different modeling strategies. In particular, performance metrics reported for the 1D-CNN and other models are intended to illustrate their potential effectiveness under constrained data conditions, rather than to represent clinically generalisable accuracy. These results primarily support methodological insights into model suitability and representation learning, and they motivate further validation on larger and independent datasets.

## 4 Discussion

### 4.1 The LSTM model

For the 90%–10% training-testing split, this classifier showed a sensitivity of 75.00%, indicating it correctly identified periapical infections in three out of every four instances. However, its specificity is much lower at 41.67%, suggesting a significant struggle in accurately classifying caries. This imbalance between sensitivity and specificity highlights that the LSTM model was biased toward recognizing periapical infections but failed to detect caries effectively. The overall performance is further reflected in the model's moderate precision of 55.56%, F1 score of 0.62, and a relatively low AUC of 0.72, indicating that while it performed reasonably well in identifying caries, its ability to distinguish between the two classes was limited.

The low performance of the LSTM model, particularly in terms of specificity when classifying caries, can be attributed to several factors. First, the LSTM's ability to capture sequential dependencies in text might be limited by the small dataset size. The model may not have enough data to learn the subtle differences between caries and periapical infections from the text alone, leading to a bias toward identifying one class (such as periapical infections) more accurately than the other. Moreover, LSTM models are typically better suited for sequential data such as time series, but they may not be the most effective for text classification tasks without appropriate preprocessing or feature engineering.

For the 5-fold cross-validation, based on Tables 4, 5, the LSTM model trained on text-only inputs exhibits modest and variable performance under 5-fold cross-validation. Overall, the results indicate that the LSTM is able to capture some discriminative patterns from the derived textual representations, but its classification capability remains limited.

The model shows a tendency toward higher sensitivity for detecting periapical infections than specificity for identifying caries, indicating an imbalance in class-wise performance. This behavior suggests that while the sequential modeling capacity of the LSTM can identify infection-related features, it is less effective at consistently distinguishing non-infection cases. The corresponding confidence intervals are relatively wide, reflecting variability across folds and highlighting the instability of the model when trained on a small dataset.

In comparison with other text-based approaches, the LSTM is consistently outperformed by both BERT and the 1D-CNN across all evaluated metrics. This suggests that the representational power of the LSTM is insufficient to fully exploit the information encoded in the derived textual features, particularly under data-limited conditions.

### 4.2 The BERT model

BERT achieved a sensitivity of 83.33%, indicating strong performance in correctly identifying periapical infection cases. However, its specificity was lower at 66.67%, suggesting a relatively higher misclassification rate for caries. While the model effectively detected periapical infections, it struggled to distinguish periapical infections from caries, which is reflected in its overall accuracy of 76.67%. The precision of 83.33% indicates that most of the positive classifications were correct, contributing to a high F1 score of 0.82, which balances precision and sensitivity. However, the AUC of 0.73 suggests moderate overall discriminative ability. This performance could stem from challenges in capturing subtle textual differences in descriptions of caries.

BERT's moderate performance may be due to reliance on textual descriptions, which can be ambiguous or inconsistent. The small dataset limits learning diverse patterns, increasing the risk of overfitting to specific linguistic variations. Additionally, medical image interpretation requires nuanced domain knowledge that text alone may not fully capture. Unlike image-based models that analyze spatial and structural features, BERT depends solely on textual abstraction, which may omit subtle visual details crucial for distinguishing between similar conditions like caries and periapical infections.

As shown in Tables 4, 5, the BERT model operating on text-only inputs demonstrates consistently stronger performance than the LSTM across all evaluation metrics under 5-fold cross-validation. These results indicate that transformer-based representations are more effective at capturing discriminative information from the derived textual features.

BERT exhibits a more favorable balance between sensitivity for detecting periapical infections and specificity for identifying caries, suggesting improved robustness in class-wise discrimination compared with recurrent models. In addition, the confidence intervals associated with BERT's performance measures are generally narrower than those of the LSTM, reflecting greater stability across cross-validation folds despite the limited dataset size.

When compared with other text-based approaches, BERT substantially improves upon the LSTM and approaches the performance of the 1D-CNN, highlighting the benefit of contextualized representations learned via self-attention. These findings suggest that the pretrained language modeling paradigm enables more effective exploitation of the structured textual representations derived from panoramic radiographs.

### 4.3 The 1D-CNN model

This net, on the other hand, demonstrated a much more balanced performance with both sensitivity and specificity at 86.67%. This indicates that the model performed well in identifying both caries and periapical infections with equal accuracy. The balance in performance is also evident in the model's high accuracy of 84.00%, precision of 86.67%, and F1 score of 0.84. Additionally, with an AUC of 0.93, the 1D-CNN model exhibited strong overall classification capability, effectively distinguishing between the two classes with a high degree of robustness.

While the 1D-CNN model demonstrated strong performance with balanced sensitivity and specificity, caution is also required when interpreting its results on the small dataset. Despite its high accuracy, the model may still face challenges in generalizing to larger or more diverse datasets. With a limited sample size, there is a risk of overfitting, where the model might learn patterns specific to the small dataset that do not hold well in broader contexts. The 1D-CNN's performance should be validated on larger datasets to ensure its robustness and reliability in real-world applications.

Tables 4, 5 show that the 1D-CNN model trained on text-only inputs achieves the strongest and most consistent performance among all evaluated approaches under 5-fold cross-validation. This indicates that convolutional modeling of the derived one-dimensional representations is particularly well suited to the characteristics of the transformed panoramic radiographic data.

The 1D-CNN demonstrates a favorable balance between sensitivity for detecting periapical infections and specificity for identifying caries, reflecting robust class-wise discrimination. Compared with recurrent and transformer-based models, its performance exhibits reduced variability across folds, as evidenced by comparatively narrower confidence intervals, suggesting greater stability under small-sample conditions.

In direct comparison with other text-based models, the 1D-CNN consistently outperforms both the LSTM and BERT across all evaluation metrics. This performance advantage suggests that local pattern extraction via convolutional filters is more effective than sequential or contextual language modeling for capturing salient features in the derived textual representations.

### 4.4 Comparisons

As shown in Table 3, the classification performance of Group 1, which includes three pretrained CNN models trained on panoramic radiographs (SqueezeNet, GoogLeNet, and AlexNet), is generally lower than that of Group 2, which consists of three text-based classifiers (LSTM, BERT, and 1D-CNN).

Among the pretrained CNN models, AlexNet achieved the highest accuracy at 66.67%, with a sensitivity of 73.33% and a specificity of 60.00%, suggesting it performed better in detecting both caries and periapical infections compared to SqueezeNet and GoogLeNet. While GoogLeNet showed relatively balanced sensitivity and specificity, it struggled with an overall accuracy of 56.67%. SqueezeNet exhibited the weakest performance, with an accuracy of only 50.00% and poor sensitivity (46.67%). The AUC values for all the pretrained CNN models remained relatively low, ranging from 0.62 to 0.73, indicating moderate classification ability.

In contrast, text-based classifiers demonstrated superior performance. The 1D-CNN model outperformed all others, achieving the highest accuracy of 84.00% with balanced sensitivity and specificity at 86.67%, along with the highest AUC of 0.93. BERT also showed strong results, with an accuracy of 76.67%, though its specificity (66.67%) was lower than its sensitivity (83.33%), suggesting a bias toward detecting caries more effectively. The LSTM model struggled, with an accuracy of 56.67%, high sensitivity (75.00%), but very low specificity (41.67%), indicating difficulty in correctly identifying periapical infections.

Overall, text-based classification outperformed image-based classification in distinguishing caries and periapical infections from panoramic radiographs, particularly with 1D-CNN and BERT achieving significantly better results than the CNN-based models. The superior performance of text-based classifiers suggests that transforming radiographic data into textual descriptions enables more effective feature extraction, reducing dependence on large annotated image datasets and complex preprocessing.

A comparison between the text-based models (LSTM, BERT, and 1D-CNN) and the pretrained image-based CNNs (SqueezeNet, GoogLeNet, and AlexNet), as reported in Tables 4, 5, reveals clear performance differences between the two modeling paradigms.

Overall, the text-based approaches demonstrate superior discriminative capability compared with the image-only CNNs. In particular, the 1D-CNN consistently outperforms all pretrained image-based models, exhibiting stronger class-wise discrimination and greater stability across cross-validation folds. This suggests that the derived one-dimensional textual representations preserve salient diagnostic information that can be more effectively exploited by convolutional architectures than by directly processing reduced-resolution images.

Among the image-based models, GoogLeNet generally performs more robustly than SqueezeNet and AlexNet, reflecting the benefit of deeper and more structured feature extraction. However, even the strongest image-based CNN remains inferior to the best-performing text-based models, indicating limitations in learning discriminative features from low-resolution panoramic images under data-scarce conditions.

The LSTM shows comparable or slightly lower performance than the pretrained CNNs, highlighting the limitations of recurrent sequence modeling for this task. In contrast, BERT consistently exceeds the performance of all image-based CNNs, underscoring the advantage of contextualized representations learned via transformer architectures. Nevertheless, both LSTM and BERT exhibit greater variability than the 1D-CNN, suggesting sensitivity to small-sample effects.

These results indicate that transforming panoramic radiographs into structured textual or signal-like representations, followed by appropriate text-based modeling, can outperform direct image-based classification using pretrained CNNs in limited-data settings. The findings further suggest that model-representation alignment plays a critical role in achieving robust performance when dataset size and image resolution are constrained.

An important methodological consideration concerns the use of reduced-resolution panoramic radiographs for the image-based models. All pretrained CNNs were trained on images downscaled by a factor of 25 to ensure computational feasibility. While this approach enabled efficient model training, it inevitably resulted in substantial loss of fine-grained anatomical detail and likely constrained the performance of image-based classifiers.

As a result, the comparative analysis presented in this study should not be interpreted as a direct or fair comparison between optimally trained image-based models and text-based approaches. Instead, the findings reflect a comparison between a degraded image-based pipeline and a text-based representation derived from the same underlying images. This distinction is critical when interpreting the observed performance differences and their implications for clinical applicability. In practice, image-based models trained on original-resolution radiographs would be expected to achieve superior diagnostic performance.

### 4.5 Training and validation

Analysis of the training and validation processes of the 1D-CNN, LSTM, and BERT models using a hold-out data partition, as shown in Figure 4, can provide insights into the classification performance of each model.

For the 1D-CNN model (Figure 4a), the training process appeared stable, with both training and validation performance improving steadily. The small gap between the solid (training) and dotted (validation) lines suggested minimal overfitting, indicating that the model generalized well to unseen data.

In the case of the LSTM model (Figure 4b), the training curve improved, but the validation curve may show more fluctuations or a larger gap, suggesting potential overfitting or difficulty in generalizing. LSTMs often struggle with smaller datasets, as they require longer sequences to fully leverage their sequential learning capabilities.

For the BERT-based model (Figure 4c), validation accuracy reached 100% while validation loss decreased, suggesting strong learning. However, this raises concerns about overfitting, as the small dataset may have led the model to memorize patterns rather than generalize. Further evaluation on independent data or a larger dataset is needed to confirm its reliability and real-world applicability.

The training and validation processes of SqueezeNet, GoogLeNet, and AlexNet exhibit overfitting, as illustrated in Figure 5. All three models reached 100% training accuracy while maintaining much lower validation accuracy. SqueezeNet struggled the most, with unstable validation accuracy and erratic validation loss, indicating poor generalization. GoogLeNet showed better training stability, but its validation accuracy fluctuated significantly, suggesting that the model memorized training patterns but failed to generalize effectively. AlexNet performed relatively better, with a more consistent validation accuracy trend, though still affected by overfitting. The high validation loss reinforced the limited ability of these pretrained CNNs to classify dental diseases from panoramic radiographs reliably.

### 4.6 Image-based text descriptions

Using ChatGPT to translate panoramic radiographs into text descriptions offers several advantages over direct classification of the images using image-based classifiers as follows.

*Ability to leverage semantic understanding:* ChatGPT can analyze and describe high-level features of radiographs in natural language, summarizing complex patterns or abnormalities in a human-readable format. These textual descriptions can encapsulate contextual information and insights that may help in downstream classification tasks.

*Simplified workflow*: Generating text descriptions directly from radiographs can simplify the workflow by reducing the preprocessing steps needed for image-based classifiers. This allows for faster and more efficient analysis, particularly in large-scale datasets.

*Scalability and resource efficiency:* Reducing image resolution (by 25 times) simplifies the computational load and storage requirements, which is particularly useful for large-scale datasets. Image-based classifiers often struggle with reduced-resolution images, as essential visual details may be lost. In contrast, ChatGPT can still infer potential abnormalities from simplified features, potentially acting as a bridge between low-quality inputs and meaningful outputs.

*Improved generalizability;* Instead of relying solely on pixel-level patterns, textual descriptions generated by ChatGPT can generalize better across variations in image quality, acquisition settings, or equipment. This approach can reduce the dependency on extensive image-based model training, which often requires high-resolution data and large labeled datasets.

*Enhanced interpretability:* Text-based outputs are inherently more interpretable to healthcare professionals than direct image classifications, making it easier to validate the results and integrate them into diagnostic workflows. Descriptions can highlight specific features, such as “possible bone loss near tooth x”, rather than providing a simple categorical label.

### 4.7 Limitations

This study has several limitations. First, the dataset is relatively small and derived from a single center, which restricts statistical power and increases the risk of overfitting, even though 5-fold cross-validation was employed. While cross-validation provides more robust performance estimates than a single train-test split, it cannot fully eliminate overfitting in data-limited settings.

The dataset may not fully capture the diversity of pediatric dental conditions across different age groups, socioeconomic backgrounds, and geographic regions. In addition, variations in image acquisition protocols, annotation quality, and clinical diagnostic criteria may introduce bias and limit the generalizability of the reported results. The absence of external validation cohorts further constrains the assessment of model robustness across different institutions and imaging environments.

Furthermore, although the proposed AI approaches demonstrate promising performance in this exploratory setting, they have not yet been validated in large-scale, prospective clinical studies. Moreover, the interpretability of the deep learning outputs requires further enhancement to support transparency, clinician trust, and clinical adoption.

In addition, the lack of longitudinal data limits the ability to assess disease progression or treatment outcomes over time. Future work involving larger, multi-center, and longitudinal datasets will be essential to address these limitations and to support the development of clinically robust AI systems.

Another limitation of this study is the absence of quantitative inter-rater agreement analysis and large-scale clinical validation. Although a qualified dentist qualitatively reviewed the generated textual descriptions to assess anatomical plausibility and adherence to the non-interpretative prompt constraints, this review was not intended to establish diagnostic accuracy. As a result, the findings should be interpreted as exploratory, reflecting methodological feasibility and relative model behavior rather than clinically validated performance.

Although the proposed framework introduces human-readable textual representations as an intermediate modality, no formal evidence is currently provided to demonstrate that these generated descriptions add diagnostic value or directly improve clinical decision-making. The textual outputs were designed to be descriptive and non-interpretative, and their role in this study is methodological rather than clinical. As such, the presence of textual descriptions should not be construed as conferring inherent clinical interpretability or diagnostic usefulness. This limitation underscores the exploratory nature of the present work. Future studies will be required to systematically evaluate whether such textual representations meaningfully support clinical reasoning, enhance diagnostic confidence, or contribute to decision-making workflows when assessed by dental professionals in real-world settings.

A semantic concern is the occasional appearance of terminology that falls outside the defined clinical scope of this study. In a small number of cases, the generated textual descriptions included terms such as “tumors” and “ankylosis”, which are not representative of the pediatric dental disease categories considered (caries and periapical infections). Although these terms were not intended as diagnoses and arose within a non-interpretative descriptive context, their presence highlights the risk of semantic drift or hallucinated content when using LLMs, even under constrained prompting. To assess the impact of this issue, the affected terms were removed and the analyses were repeated. No meaningful changes in model performance were observed, indicating that the reported results were not driven by the inclusion of such terminology.

### 4.8 Future work

Future research will focus on addressing the identified limitations and enhancing clinical applicability. Expanding the dataset size, including additional disease categories, through multi-center collaborations will be essential for improving robustness, fairness, and generalisability across diverse populations. In particular, large-scale and multi-center datasets will enable robust external validation and reduce dataset-specific bias.

Strengthening clinical validation will be a priority in future work through systematic expert evaluation involving multiple dental professionals. This will include structured qualitative assessments supported by questionnaires to evaluate the perceived clinical relevance, clarity, and usefulness of the generated textual representations, alongside quantitative inter-rater reliability analysis to assess consistency across expert assessments. Together, these measures will enable a more rigorous evaluation of the accuracy, consistency, and potential clinical value of the proposed approach.

Together, these evaluations will enable a more robust determination of whether the proposed textual representations meaningfully align with clinical findings and whether they provide added value in real-world dental practice, beyond their current role as an intermediate representation for exploratory modeling.

In addition, incorporating multimodal data–such as radiographic images, clinical records, behavioral indicators, and temporal information–may further enhance predictive performance and enable early detection of disease onset or progression. The development of explainable AI frameworks and calibration methods will also be prioritized to improve interpretability and clinician confidence. Pilot clinical studies in pediatric dental settings will be necessary to evaluate workflow integration, usability, and impact on diagnostic efficiency.

Future research will also include benchmarking the proposed text-based and signal-derived approaches against image-based models trained on original-resolution radiographs. This will enable a more clinically representative and methodologically balanced comparison, clarifying the relative advantages and limitations of each modeling paradigm. Such evaluations will be essential for determining the conditions under which alternative representations may offer practical benefits and for guiding the translation of these methods into real-world clinical workflows.

Future studies will also include formal ablation analyses to more systematically evaluate the contribution of individual components within the proposed framework. Such analyses will examine the impact of representation choices, model architectures, and key design decisions by selectively removing or modifying components under controlled conditions. Conducting these ablation studies on larger datasets will be essential to obtain statistically reliable insights, reduce overfitting effects, and better understand the factors driving model performance and robustness.

Regarding model selection considerations, recent vision transformer (ViT) architectures (Han et al., 2023; Saha and Xu, 2025; Wang et al., 2025) were considered conceptually but were not included in the present experiments. Vision transformers generally require substantially larger datasets or extensive pretraining and fine-tuning to achieve stable and reliable performance. Given the limited dataset size and the exploratory focus of this study, incorporating ViT-based models would likely introduce greater performance variability and increase the risk of overfitting. Accordingly, established pretrained CNNs were selected as image-based baselines to provide a controlled comparison with the proposed text-based approaches. Future work will include systematic evaluation of ViT models when larger datasets become available, enabling a more comprehensive assessment of their potential advantages in this application.

### 4.9 Clinical implications

The proposed AI-based approach offers significant potential for improving pediatric dental care. By assisting clinicians in early detection of caries, developmental anomalies, and periodontal conditions, it may facilitate timely interventions and reduce the burden of untreated oral diseases in children. Automated image analysis and risk stratification could support preventive care programs, particularly in resource-limited or community-based settings. Furthermore, integrating such AI tools into routine dental examinations may enhance diagnostic consistency, optimize treatment planning, and improve patient engagement through personalized education. The adoption of AI in pediatric dentistry could transform preventive strategies and promote long-term oral health outcomes.

From a clinical perspective, it should be pointed out that the adopted experimental framing highlights a potential role for text-based or signal-derived representations in scenarios where image quality, spatial resolution, storage capacity, or data transmission bandwidth are limited. Such conditions may arise in low-resource clinical settings, tele-dentistry applications, or when working with legacy imaging systems. In these contexts, structured textual abstractions derived from radiographs may preserve diagnostically relevant information even when image fidelity is compromised. Nevertheless, this use case should be viewed as complementary rather than substitutive, as high-resolution image-based analysis remains the preferred approach for routine clinical decision-making.

## 5 Conclusions

This study demonstrates the potential of using a large language model to translate panoramic radiographs into textual descriptions for dental disease classification using deep learning. By leveraging text-based classification instead of direct image-based analysis, the approach eliminates the need for complex image preprocessing, such as segmentation, and allows models like 1D-CNN to process text-based descriptions efficiently. Compared to traditional image-based classifiers, this method offers greater interpretability and accessibility, particularly in settings where high-resolution imaging or expert annotations are limited.

Beyond dental disease classification, this approach has broader applications in medical imaging, where textual descriptions can be used for AI-driven diagnosis across radiology, dermatology, and pathology. Future research should focus on optimizing text generation from images, ensuring that descriptions capture clinically relevant features with high fidelity. Additionally, investigating hybrid models that combine text-based and image-based features may enhance classification accuracy and generalizability. Expanding datasets, improving domain-specific language models, and incorporating external clinical knowledge into text-based AI models will be crucial for advancing AI-assisted diagnostics in healthcare.

## Data Availability

Publicly available datasets were analyzed in this study. This data can be found here: Children's Dental Panoramic Radiographs Dataset for Caries Segmentation and Dental Disease Detection. Figshare: https://doi.org/10.6084/m9.figshare.c.6317013.v1.
